# iHOT-12 item analysis: correlations between individual items and overall score within and across time points

**DOI:** 10.1093/jhps/hnaf004

**Published:** 2025-02-20

**Authors:** Matthew Yuro, Robroy Martin, Andrew B Wolff, Shane Nho, Thomas Wuerz, Geoff Van Thiel, John Christoforetti, John P Salvo, Dean Matsuda, Dominic S Carreira

**Affiliations:** Peachtree Orthopedics, 3200 Downwood Circle NW #700, Atlanta, GA 30327, United States; Duquesne University, 600 Forbes Ave, Pittsburgh, PA 15282, United States; Washington Orthopaedics and Sports Medicine, 2021 K Street NW, Washington DC 20006, United States; Midwest Orthopaedics at Rush, 1611 W Harrison St, Chicago, IL 60612, United States; Boston Bone and Joint Institute, 840 Winter St, Waltham, MA 02451, United States; Ortho Illinois, 324 Roxbury Rd, Rockford, IL 61107, United States; The Centers for Advanced Orthopaedics, 6710A Rockledge Drive, Bethesda, MD 20817, United States; Rothman Orthopaedic Institute, 999 Route 73 N, Marlton, NJ 08053, United States; Premier Hip Arthroscopy, 13160 Mindanao Way, Marina Del Rey, CA, United States; Peachtree Orthopedics, 3200 Downwood Circle NW #700, Atlanta, GA 30327, United States

## Abstract

The main aim of this study was to analyze correlations between individual international hip outcome tool 12 (iHOT-12) items and overall iHOT-12 score within and across timepoints. A retrospective multicenter hip arthroscopy registry was queried for patients from January 2014 to October 2023 with completed iHOT-12 reports. Pearson coefficient analysis was used to identify correlations between individual iHOT-12 items and overall iHOT-12 score at each timepoint and between preoperative individual iHOT-12 items and postoperative overall iHOT-12 scores. Validity, reliability, and responsiveness of the iHOT-12 were analyzed at each timepoint. Within timepoints, correlations ranged in strength from fair (preoperative maintain fitness level, *r* = 0.52, *P* < .00001) to excellent (5-year hip pain after activity, *r* = 0.93, *P* < .00001). Correlations increased in strength over time. Across timepoints, correlations ranged in strength from no correlation (2-year maintain fitness level, *r* = −0.001, *P* = 0.94) to poor (6-month pushing or lifting heavy objects, *r* = 0.31, *P* < .00001). Correlations decreased in strength over time. No ceiling or floor effects were exhibited. The Cronbach alpha for the baseline, 6-month, 1-year, 2-year, and 5-year timepoints were 0.87, 0.96, 0.95, 0.96, and 0.97, respectively. Cohen’s *d* values at 6 months, 1 year, 2 years, and 5 years were 1.19, 1.43, 1.71, and 1.58, respectively. Within timepoints, hip pain after activity had the strongest correlations to overall iHOT-12 score. Across timepoints, correlations between preoperative individual iHOT-12 items and postoperative overall iHOT-12 score were poor and weakened over time, suggesting similar long-term postoperative iHOT-12 scores among both high- and low-scoring preoperative patients. The iHOT-12 demonstrated good validity, reliability, and responsiveness at all timepoints.

## Introduction

Patient-reported outcome measures (PROMs) are valuable clinical tools used to evaluate patient functioning preoperatively, to assess outcomes, and define surgical success, and they have seen a recent increase in use within the field of hip arthroscopy [1, 2]. Several PROMs have been developed to measure the success of hip arthroscopy [3–6]. Many existing PROMs were designed for older and less active cohorts and experienced ceiling effects when applied to younger active cohorts, those more likely to undergo hip arthroscopy [7]. The international hip outcome tool 33 (iHOT-33) sought to measure outcomes of hip surgery taking into account the quality of life of younger patients [3]. For many providers, a 33-item survey was too long and impractical for clinical use, so a shorter version called the iHOT-12 was created using 12 items from the original survey that accounted for 96–99% of the total variation of the full score [8]. Recent systematic reviews have reported the iHOT-12 to be as thoroughly validated as the Hip Outcome Score (HOS) and Hip And Groin Outcome Score (HAGOS) with regard to its psychometric properties and score interpretation following hip arthroscopy [2, 9]. However, little is known about the ability of iHOT-12 in predicting outcomes at mid-term and long-term postoperative timepoints.

Over the past decade, several studies have verified the psychometric properties of the iHOT-12, and it has been validated in several languages [2, 8, 10–13]. Many previous validation studies analyzed the iHOT-12 taken at a single timepoint. When comparison timepoints were used to test consistency over time, they were often spaced only weeks apart [3, 10, 11]. Analyses of the iHOT-12 taken by a surgical cohort at the 6-month and 1-year postoperative timepoints have been conducted and used as a comparison to validate other PROMs [14–16]. However, these studies are limited in scope by short-term outcomes. There is little literature analyzing the psychometric properties of the iHOT-12 at mid-term and long-term postoperative timepoints and especially using individual item responses from iHOT-12 scores to interpret and predict outcomes.

Two-year and five-year iHOT-12 outcomes have been used to determine minimal clinically important difference (MCID), substantial clinical benefit (SCB), and patient acceptable symptomatic state (PASS) [17, 18]. Many studies have concluded values for MCID, SCB, and PASS are helpful at interpreting iHOT-12 scores at a particular time point, and recent literature suggests these values may also have predictive value. Failing to meet MCID and PASS at 1-year postoperatively has been shown to increase risk of a subsequent hip operation at minimum 2 years postoperatively [19]. While MCID, SCB, and PASS are interpreted in conjunction with iHOT-12 total score, no previous research has analyzed the predictive capability of individual iHOT-12 items.

The present study aims to analyze individual iHOT-12 items to test for predictive capability of overall function at a particular timepoint as well as across timepoints. It is hypothesized that certain items would be more predictive of overall function than others within timepoints and across preoperative to postoperative timepoints. This information would be valuable to clinicians seeking to understand patient risk and predict success following hip arthroscopy, given a particular set of symptoms. An additional purpose of this study is to analyze and compare psychometric properties of the iHOT-12 at the preoperative, 6-month, 1-year, 2-year, and 5-year timepoints. Additionally, it is hypothesized that the iHOT-12 would be valid, reliable, and responsive at all timepoints.

## Methods

### Patient selection

A retrospective review on a prospectively maintained database from a multicenter hip arthroscopy study group was performed. Inclusion criteria were patients undergoing arthroscopic hip surgery between January 2014 and October 2023 who did not respond to nonoperative management (physical therapy, activity modification, oral anti-inflammatory drugs, or intraarticular cortisone injection) for 6 months. Indications for surgery included but were not limited to labral tear, Femoroacetabular Impingement (FAI), and atraumatic microinstability. Eight surgeons from 8 medical centers with a minimum of 9 years of experience and 150 hip arthroscopy surgeries performed per year were included in this study. All patients were evaluated by the senior surgeons at their respective medical centers. Exclusion criteria were patients with worker’s compensation insurance and patients with incomplete patient-reported outcome forms. The commercial data collection services Outcomes Based Electronic Research Database (Columbia, MO, USA) and PatientIQ (Chicago, IL, USA) were used for data collection. Some patients included in the present study may overlap with previous studies from our multicenter organization using the same patient registry.

### Functional outcome evaluation

Demographic data including patient sex, age, operative laterality, and body mss index (BMI) were collected preoperatively. The iHOT-12 PROM was administered to all patients preoperatively, and at 6 months, 1 year, 2 years, and 5 years postoperatively. The iHOT-12 consists of four domains: symptoms and functional limitations, sport and recreational activities, job-related concerns, and social, emotional, and lifestyle concerns [18]. These domains are measured by 12 items: hip and groin pain, difficulty getting up and down, walking long distances, grinding in the hip, pushing or lifting heavy objects, changing directions, hip pain after activity, carrying children, sexual activity, awareness of disability, ability to maintain fitness level, and health-related distraction. Each item is scored using a visual analog scale from 1 to 100, with a score of 100 representing the best function and least among of symptoms.

### Statistical analysis

Sample and instrument characteristics were examined using mean, standard deviation, proportion, and correlation as appropriate. Pearson coefficient analysis was used to identify correlations between individual iHOT-12 items and overall iHOT-12 score at each timepoint. Secondly, Pearson coefficient analysis was also used to identify correlations between individual iHOT-12 items at the preoperative timepoint and overall iHOT-12 scores at postoperative timepoints. Lastly, Pearson coefficient analysis was used to identify correlations between preoperative overall iHOT-12 score and overall iHOT-12 score at postoperative timepoint. Correlation was defined as follows: excellent, greater than 0.80; very good, 0.71 to 0.80; good, 0.61 to 0.70; fair, 0.41 to 0.60; or poor, 0.21 to 0.40 [20]. Student’s *t*-distributions were used to determine the statistical significance of correlations.

Validity, reliability, and responsiveness of the iHOT-12 were analyzed at each timepoint. Instrument range was used to evaluate validity. Ceiling and floor effects were defined by having any percentage of patients ≥15% of the study population achieving the maximum or minimum score [21, 22]. To evaluate reliability, internal consistency was analyzed at each timepoint via the Cronbach alpha [23]. Cronbach alpha ranges from 0 to 1, and a value of ≥ 0.70 was defined as adequate [24]. Responsiveness was evaluated at each timepoint via effect size, or Cohen *d*, and via standardized response mean (SRM) [25]. Cohen *d* measures the magnitude of the preoperative-to-postoperative change in relation to the amount of variability in the scores [14]. Cohen *d* was defined as follows: very good, greater than 1.31; large, 0.81 to 1.3; medium, 0.51 to 0.80; small, 0.2 to 0.5 [26]. The SRM estimates an outcome measure’s ability to detect change over time. SRM was defined as follows: large, greater than 0.8; moderate, 0.5–0.8; and low, 0.2–0.5[27].

Descriptive statistics for all continuous variables are reported as means with standard deviations, and frequency statistics are reported for all noncontinuous variables. Paired-sample *t*-tests were used to compare preoperative and postoperative PROMs. Statistical significance for all analyses was set at α ≤ 0.05. All statistical analyses were performed using SPSS, Version 26 (IBM Corp., Armonk, NY, USA).

## Results

### Patient demographics

A total of 7689 patients underwent hip arthroscopy between January 2014 and October 2023. Of these patients, 178 were excluded due to worker’s compensation status and 4018 had missing or incomplete PROMs. A total of 3493 patients remained with complete PROMs. These patients completed a total of 6776 iHOT-12 surveys; 3170 at the preoperative timepoint, 554 at 6-month, 797 at 1-year, 1114 at 2-year, and 532 at the 5-year postoperative timepoint. Of the included patients, 66.3% were female and the right hip was operated on in 54.6% cases. The average age and BMI were 34.31 ± 13.21 years and 24.92 ± 9.89 kg/m [2], respectively (Table 1).

**Table 1.** Baseline patient characteristics.

Total patients	3493
Sex ^a^	
Female	66.3
Laterality^a^	
Right	54.6
Age, years^b^	34.31 ± 13.21
BMI^c^	24.92 ± 9.89

Characteristics of sampled patients at the preoperative timepoint. .

a Values for sex, laterality, and age <35 years are reported as percentages (%)..

b Age is reported in years.

c BMI is reported in kg/m^2^.

### Clinical outcomes analysis

There were statistically significant improvements in iHOT-12 score from the preoperative timepoint to all postoperative timepoints, including 6 months (37.4 ± 19.5 vs. 65.6 ±27.4, *P* < .00001), 1 year (36.3 ± 18.4 vs. 69.4 ± 26.8, *P* < .00001), 2 years (37.6 ± 18.0 vs. 74.9 ± 25.1, *P* < .00001), and 5 years (37.3 ± 18.0 vs. 73.1 ± 26.4, *P* < .00001) (Table 2).

**Table 2.** iHOT-12 clinical outcomes.

Timepoint	Overall iHOT-12 score	*P*-value ^a^
Preoperative	38.4 ± 18.7	-
6 months	65.6 ± 27.4	<.00001*
1 year	69.4 ± 26.8	<.00001*
2 years	74.9 ± 25.1	<.00001*
years	73.1 ± 26.4	<.00001*

Average iHOT-12 score at the preoperative and 6 month 1-year, 2-year, and 5-year postoperative timepoints. The iHOT-12 measures health-related quality of life and changes after treatment in young, active patients with hip disorders.

a *P*-values are in comparison to preoperative overall iHOT-12 score for each respective postoperative cohort.

* Statistically significant (*P* ≤ .05).

### Correlation analysis

#### Within timepoints

**Figure 1.**
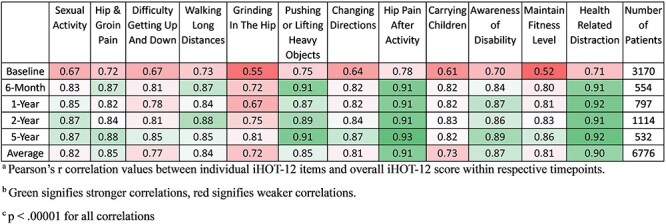
Within timepoint correlations. (a) Pearson’s *r* correlation values between individual iHOT-12 items and overall iHOT-12 score within respective timepoints. (b) Green signifies stronger correlations, red signifies weaker correlations. (c) *P* < .00001 for all correlations. The iHOT-12 survey consists of 12 individual items which contribute to overall iHOT-12 score. Pearson’s *r* correlation analyses were conducted between 12 individual item scores and the overall iHOT-12 score within respective timepoints. *R*-values describe the strength of the correlation between two variables. Correlation was defined as follows: excellent, greater than 0.80; very good, 0.71 to 0.80; good, 0.61 to 0.70; fair, 0.41 to 0.60; or poor, 0.21 to 0.40.20 Green signifies stronger correlations, red signifies weaker correlations. Student’s *t*-distributions were used to determine the statistical significance of correlations. All correlations were statistically significant (*P* < .00001).

Within timepoint correlations are summarized in Fig. 1. All individual iHOT-12 items were significantly correlated with the overall iHOT-12 scores of their respective timepoints (*P* < .00001 for all correlations). Correlations ranged in strength from fair (preoperative maintain fitness level, *r* = 0.52) to excellent (5-year hip pain after activity, *r* = 0.93). Individual item correlations with overall iHOT-12 score increased with postoperative time. Items at the preoperative timepoint had an average correlation with overall iHOT-12 score of *r* = 0.67, while items at the 5-year postoperative timepoint had an average correlation with overall iHOT-12 score of *r* = .87.

#### Across timepoints

**Figure 2.**
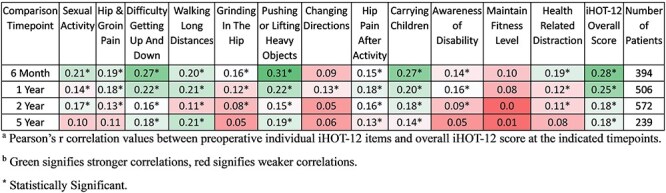
Across timepoint correlations. (a) Pearson’s *r* correlation values between preoperative individual iHOT-12 items and overall iHOT-12 socre at the indicated timepoings. (b) Green signifies stronger correlations, red signifies weaker correlations. The iHOT-12 survey consists of 12 individual items which contribute to overall iHOT-12 score. Pearson’s *r* correlation analyses were conducted between 12 individual item scores at the preoperative timepoint and the overall iHOT-12 score at indicated postoperative timepoints. *R*-values describe the strength of the correlation between two variables. Correlation was defined as follows: excellent, greater than 0.80; very good, 0.71 to 0.80; good, 0.61 to 0.70; fair, 0.41 to 0.60; or poor, 0.21 to 0.40.20 Green signifies stronger correlations, red signifies weaker correlations. Student’s *t*-distributions were used to determine the statistical significance of correlations. Asterisk indicates statistical significance.

Across timepoint correlations are summarized in Fig. 2. Correlations were analyzed between preoperative individual iHOT-12 items and overall iHOT-12 scores at the 6-month, 1-year, 2-year, and 5-year postoperative timepoints. Correlations ranged in strength from no correlation (2-year maintain fitness level, *r* = −0.001, p = 0.94) to poor (6-month pushing or lifting heavy objects*, r* = 0.31, *P* < .00001). No correlations met the threshold to be classified as excellent, very good, good, or fair. Correlations decreased in strength over time. There were 10 statistically significant correlations between individual preoperative iHOT-12 items and overall iHOT-12 score at the 6-month postoperative timepoint, and there were 5 statistically significant correlations between individual preoperative iHOT-12 items and overall iHOT-12 score at the 5-year postoperative timepoint (Table 3).

**Table 3.** Statistical significance of across timepoint correlations.

	Statistically significant correlations^a^	Statistically nonsignificant correlations
6 Months	10	2
1 Year	11	1
2 Years	10	2
5 Years	5	7

The iHOT-12 survey consists of 12 individual items that contribute to overall iHOT-12 score. Pearson’s *r* correlation analyses were conducted between 12 individual item scores at the preoperative timepoint and overall iHOT-12 score at postoperative timepoints. Student’s *t*-distributions were used to determine the statistical significance of correlations.

Number of statistically significant correlations per timepoint out of 12 possible correlations are listed.

a While determined to be statistically significant, all correlations are considered poor (*r* < 0.40) [20].

Preoperative overall iHOT-12 score had a poor positive correlation with 6-month overall iHOT-12 score (*r* = 0.28, *P* < .00001), and this correlation weakened at 1 year, 2 years, and 5 years (*r* = 0.25, *P* < .00001; *r* = .18, *P* = .00001; *r* = 0.18, *P* = .006, respectively).

### Validity analysis

Instrument range was used to evaluate iHOT-12 validity (Table 4). No ceiling or floor effects were exhibited at any timepoint for overall iHOT-12 score. The percentage of patients achieving maximum 100% scores increased with time, ranging from 0.19% preoperatively to 5.45% at 5 years postoperation. The percentage of patients achieving minimum 0% scores remained very low at all timepoints, ranging from 0.27% at 2 years postoperation to 1.5% at 5 years postoperation. At the preoperative timepoint, one individual item displayed a ceiling effect (“carrying children”) and four individual items displayed floor effects (“changing directions,” “awareness of disability,” “maintain fitness level,” and (“health-related distraction”). At the 6-month and 1-year postoperative timepoints, five individual items displayed ceiling effects (“difficulty getting up and down,” “walking long distances,” “grinding in the hip,” “pushing or lifting heavy objects,” and “carrying children.” At the 1-year postoperative timepoint, one individual item displayed a floor effect (“maintain fitness level”). At the 2-year postoperative timepoint, nine individual items displayed ceiling effects (“sexual activity,” “difficulty getting up and down,” “walking long distances,” “grinding in the hip,” “pushing or lifting heavy objects,” “changing direction,” “carrying children,” “maintain fitness level,” and “health-related distraction.” At the 5-year postoperative timepoint, all 12 items displayed ceiling effects.

**Table 4.** iHOT-12 psychometric properties.

	Baseline	6 Months	1 Years	2 Years	5 Years
Range^a^					
Ceiling effect (%)	0.19	1.08	2.63	3.95	5.45
Floor effect (%)	0.28	0.54	0.38	0.27	1.5
Cronbach alpha^b^	0.87	0.96	0.95	0.96	0.97
Cohen *d*^c^	-	1.19	1.44	1.71	1.58
Standardized mean response	-	0.98	1.16	1.32	1.22

Validity, reliability, and responsiveness of the iHOT-12 were analyzed at each timepoint.

a Ceiling and floor effects describe the percent of patients achieving a maximum or minimum overall iHOT-12 score at respective timepoints. Effects ≥15% are considered statistically significant [21, 22].

b Cronbach alpha measures internal consistency and can range from a value of 0 to 1. Values at or above 0.70 are considered adequate [24].

c Responsiveness was evaluated at each timepoint via effect size (Cohen’s *d*) and via standardized response mean (SRM). Cohen’s *d* scores from 0.81 to 1.3 are considered large and scores greater than 1.31 are considered very good [26].

d SRM values above 0.8 are considered large [27].

### Reliability analysis

To evaluate reliability, internal consistency was analyzed at each timepoint via the Cronbach alpha (Table 4). The Cronbach alpha for the baseline, 6-month, 1-year, 2 year, and 5-year timepoints were 0.87, 0.96, 0.95, 0.96, and 0.97, respectively. All Cronbach alpha values were considered adequate, indicating good internal consistency reliability at all timepoints.

### Responsiveness analysis

Responsiveness was evaluated at each timepoint via effect size, or Cohen *d*, and via SRM (Table 4). Effect size and SRM measure responsiveness between preoperative overall iHOT-12 score and overall iHOT-12 score at postoperative timepoints. Cohen *d* values were 1.19 at 6 months, 1.43 at 1 year, 1.71 at 2 years, and 1.58 at 5 years. Effect size was considered large at 6 months and very good at 1 year, 2 years, and 5 years. SRM values were 0.98 at 6 months, 1.16 at 1 year, 1.33 at 2 years, and 1.22 at 5 years. SRM values indicated large responsiveness at all timepoints.

## Discussion

Within preoperative and 6-month, 1-year, 2-year, and 5-year postoperative timepoints, all 12 individual iHOT-12 items had statistically significant positive correlations with overall iHOT-12 score. The psychometric properties of the iHOT-12 PROM have been studied and verified several times, providing strong evidence that each individual item in the survey contributes important information to overall hip function [2, 8, 10–13]. However, the present correlation analysis demonstrates that responses to certain items better reflect overall hip function. The overall iHOT-12 score is calculated as the mean of the 12 individual questionnaire items [8]. Therefore, while all items contribute to overall score with equal weight, individual items with low variability and means close to the overall score correlate better to overall score.

The “Hip Pain After Activity” item of the iHOT-12 questionnaire had the strongest correlation to overall iHOT-12 within timepoints, with an excellent average correlation of *r* = 0.91. This may be meaningful to clinicians seeking to better understand patient symptoms. At an initial visit, asking a patient to rate their hip pain after activity on a scale of 1–10 can provide useful information. Since this question is strongly correlated to overall hip function, responses close to the maximum pain value of 10 may prompt a provider to further evaluate hip-related symptoms and consider impairment of the patient’s overall hip function. Previous research has shown that failing to reach threshold values for overall iHOT-12 score increases risk of needing a subsequent operation following a hip arthroscopy [19]. The present study sought to use individual item responses from preoperative iHOT-12 questionnaires to predict overall iHOT-12 scores at postoperative timepoints. Comparing preoperative individual iHOT-12 items with postoperative overall iHOT-12 scores, no strong correlations were found (Fig. 2). Furthermore, poor correlations between preoperative individual iHOT-12 items and short-term postoperative iHOT-12 overall score appeared to weaken over time. While the strongest correlation between preoperative individual iHOT-12 item and 6-month overall iHOT-12 score was *r* = 0.31 (“pushing or lifting heavy objects”), the strongest correlation at 5 years postoperatively had reduced to *r* = 0.21 (“walking long distances”). Similarly, the weakest correlation between preoperative individual iHOT-12 item and postoperative overall iHOT-12 decreased from *r* = 0.09 at 6 months (“changing directions”) to 0.01 at 5 years (“walking long distances”). Generally, preoperative iHOT-12 item scores were not able to provide strong predictions of long-term hip functioning following hip arthroscopy. However, with increased postoperative time, patients tend to score similarly on the iHOT-12, regardless of preoperative score. While this undermines any long-term predictive capability of the iHOT-12, it demonstrates equally successful long-term outcomes for high- and low-scoring preoperative iHOT-12 patients.

Evidence supporting the validity of the iHOT-12 PROM was found at all timepoints from 6 months to 5 years. No significant ceiling or floor effects were exhibited for overall iHOT-12 score preoperative or at 6-month, 1-year, 2-year, and 5-year postoperative timepoints. As expected, the number of patients achieving a maximum score increased as follow-up time increased. Previous validity analyses of the iHOT-12 have shown mixed range results. A study of 171 patients concluded that the iHOT-12 had a ceiling effect at 1-year postoperatively [16]. However, another iHOT-12 analysis at 1-year postoperatively showed no floor or ceiling effects among 124 patients [14]. The present study concluded that 2.63% of 797 patients achieved a maximum score of 100 on the iHOT-12 at the 1-year postoperative timepoint, which does not meet the 15% threshold of defining a ceiling effect [21]. Although ceiling and floor effects were not observed in overall iHOT-12 scores, several individual items demonstrated ceiling and floor effects at multiple timepoints. For example, 39% of respondents preoperatively had the minimum possible response to “maintain fitness level,” and 52% of 5-year postoperative respondents had the maximum possible response to “carrying children.” As the overall iHOT-12 score is calculated from the mean visual analog scale score for the individual questionnaire items, range limitations with individual items may result in accuracy issues for overall iHOT-12 score [8].

The iHOT-12 PROM was found to be reliable and responsive at all timepoints analyzed. Cronbach alpha was used to evaluate reliability, and values ranged from 0.87 preoperatively to 0.97 at 5-year postoperatively. All values were well above the adequate value of 0.70 [23]. Similarly, high Cronbach alpha values have been reported in previous psychometric analyses of the iHOT-12 [11, 12, 28]. Responsiveness was evaluated via effect size, or Cohen *d*, and via SRM. Effect size can allow for a more useful clinical understanding of differences between groups than *P*-value. With sufficiently high patient populations, clinically insignificant differences between groups may still be considered statistically significant. Effect size takes population size into account, and it can be interpreted as a percentage of the standard deviation [29]. Therefore, an effect size of 0.8 means that the difference between two groups is 80% of the standard deviation of the group, and this is considered large regardless of population size. In the present study, Cohen *d* values ranged from 1.19 at 6 months postoperatively to 1.71 at 2 years postoperatively. These effect sizes are considered large and support previously reported 1-year postoperative iHOT-12 effect sizes of *d *= 1.77 and *d *= 1.59 [14, 16]. The SRM is an additional index of effect size. Whereas Cohen *d* utilizes pooled standard deviation between pre- and postoperative scores to calculate effect size, SRM uses the simple standard deviation of the difference in scores [27]. This allows for an additional metric to estimate responsiveness. The SRM values in the present study ranged from 0.98 at 6 months postoperatively to 1.33 at 2 years postoperatively, all falling within the classification of large responsiveness. In addition to supporting the conclusions of short- and mid-term analyses, the present study demonstrates that the iHOT-12 questionnaire continues to be responsive and reliable at long-term timepoints.

## Limitations

This study has several limitations. First, sample size is not consistent across all timepoints. The present study included 3170 preoperative iHOT-12 questionnaires, and this number reduced to 532 at 5 years postoperatively. Some patients had not yet reached postoperative timepoints, and some had failed to complete postoperative forms. Furthermore, as all forms were voluntary for patients to complete, a selection bias may have resulted in certain patient populations being excluded from the study. Additionally, this study included all patients from a multicenter hip registry who underwent hip arthroscopy. Indications for surgery included but were not limited to labral tear, FAI, and atraumatic microinstability. It is possible that if broken down by procedure, certain patient populations could have different results. Finally, this study contained patients from eight different surgeons. Although this increases the generalizability of the results, unaccounted differences in recovery protocol could have affected outcomes.

## Conclusion

Within timepoints, the iHOT-12 had many strong correlations between individual items and overall score, with hip pain after activity being most predictive of overall hip function. Across timepoints, correlations were poor and overall hip function could not be predicted based on a singular item response from a previous questionnaire. The iHOT-12 demonstrated good validity, reliability, and responsiveness at preoperative, 6-month, 1-year, 2-year, and 5-year timepoints.

## Data Availability

Data are available on request. The data underlying this article will be shared on reasonable request to the corresponding author.
